# Highly Pathogenic Avian Influenza A(H5N1) Outbreaks in West Java Indonesia 2015–2016: Clinical Manifestation and Associated Risk Factors

**DOI:** 10.3390/microorganisms7090327

**Published:** 2019-09-06

**Authors:** Desniwaty Karo-karo, Diyantoro, Eko Sugeng Pribadi, Fransiscus Xaverius Sudirman, Sussi Widi Kurniasih, Sukirman, Iin Indasari, David Handojo Muljono, Guus Koch, Jan Arend Stegeman

**Affiliations:** 1 Department of Farm Animal Health, Faculty of Veterinary Medicine Utrecht University, 3584 CL Utrecht, The Netherlands; 2 Centre for Diagnostic Standard of Indonesian Agricultural Quarantine Agency, Ministry of Agriculture, Jakarta 13220, Indonesia; 3 Center for Tropical Animal Studies, Institute of Research and Community Empowerment, Bogor Agricultural University, Bogor 16129, Indonesia; 4 ProLab Diagnostic Laboratory, PT. Sierad Produce, Tbk, Bogor 16340, Indonesia; 5 Livestock and Animal Health Agency of District Subang, Subang 41214, Indonesia; 6 West Java Province Animal Health Agency, Bandung 40135, Indonesia; 7 Eijkman Institute for Molecular Biology, Jakarta 10430, Indonesia; 8 Wageningen Bioveterinary Research, 8221 RA Lelystad, The Netherlands

**Keywords:** HPAI (H5N1), risk factors, West Java, outbreak investigation, case-control

## Abstract

Knowledge of outbreaks and associated risk factors is helpful to improve control of the Highly Pathogenic Avian Influenza A(H5N1) virus (HPAI) in Indonesia. This study was conducted to detect outbreaks of HPAI H5N1 in endemically infected regions by enhanced passive surveillance, to describe the clinical manifestation of these outbreaks and identify associated risk factors. From November 2015 to November 2016, HPAI outbreak investigations were conducted in seven districts of West Java. In total 64 outbreaks were confirmed out of 75 reported suspicions and outbreak characteristics were recorded. The highest mortality was reported in backyard chickens (average 59%, CI_95%_: 49–69%). Dermal apoptosis and lesions (64%, CI_95%_: 52–76%) and respiratory signs (39%, CI_95%_: 27–51%) were the clinical signs observed overall most frequently, while neurological signs were most frequently observed in ducks (68%, CI_95%_: 47–90%). In comparison with 60 non-infected control farms, the rate of visitor contacts onto a farm was associated with the odds of HPAI infection. Moreover, duck farms had higher odds of being infected than backyard farms, and larger farms had lower odds than small farms. Results indicate that better external biosecurity is needed to reduce transmission of HPAI A(H5N1) in Indonesia.

## 1. Introduction

Ever since its first notification in Hong Kong in the late 90s [1,2], Highly Pathogenic Avian Influenza A (HPAI) viruses of subtype H5N1 have caused numerous outbreaks in poultry worldwide and hundreds of (mostly fatal) cases in humans [3,4]. In addition, because the virus became endemic to the poultry populations of several countries in Asia and in Egypt there was ample opportunity for reassortment with other AI viruses, which has resulted in a multitude of HPAI virus clades that have subsequently circulated globally [5,6,7,8,9]. As long as there are endemically infected regions, new reassortants will arise and pose a threat to global poultry and wild bird populations and to humans and other mammals such as pigs and cats.

To control the spread of HPAI among poultry flocks, biosecurity, early detection and depopulation of infected flocks are all of crucial importance. In addition, if vaccination is applied, (i) high-quality vaccines should be used based on viruses that are antigenically closely related to circulating field strains; (ii) vaccination coverage should be high; and (iii) vaccination should be accompanied by adequate surveillance to not result in silent spread of the virus [10]. Information on risk factors associated with outbreaks in the countries endemically infected with HPAI may be helpful to support control programs to eliminate the virus from such regions in the long run [11,12].

Since 2003, HPAI A(H5N1) infections have been endemic to the Indonesian poultry population [13]. Collector houses and live bird markets play an important role in the spread of infection [14,15,16]. Nevertheless, knowledge on risk factors for HPAI on poultry farms is also important for the control of the infection. Studies into factors associated with HPAI H5N1 outbreaks in Indonesian poultry have been previously reported, but they used secondary data, defined outbreaks on clinical diagnosis or the outcome of non-validated rapid tests only or did not use controls [17,18,19]. In addition, to our knowledge no risk factor studies concerning the currently circulating virus clade 2.3.2.1c [20,21] have been published. Consequently, a study describing the characteristics of outbreaks caused by currently circulating HPAI A(H5N1) and quantifying associated risk factors is valuable, in particular because the Indonesian poultry sector is expanding due to the growing demand for poultry products.

West Java is the province in Indonesia which has been affected most severely by HPAI H5N1 outbreaks, because it has the highest poultry density, intensive poultry trading and a poultry sector consisting of various types of poultry, including ducks. The aims of this study were to detect outbreaks of HPAI H5N1 in an endemically infected region by enhanced passive surveillance, describe the mortality, morbidity and clinical manifestation of these outbreaks and identify associated risk factors.

## 2. Materials and Methods

### 2.1. Poultry Farming in Indonesia

According to the classification used by the Food and Agricultural Organization of the United Nations (FAO), the poultry production in Indonesia can be classified in four sectors [22,23]. Sector 1 farms are associated with high biosecurity and industrial integrated production. The chickens are brought to slaughterhouses and products are commercially marketed. Sector 2 farms have moderate to high biosecurity with medium to large scale production. Birds or products are sold via poultry markets or slaughterhouses. Sector 3 farms are of low biosecurity and small to medium size. Birds and products are usually sold through live bird markets. Backyard chicken farming is classified as poultry sector 4 and includes the majority of poultry farms in Indonesia. It is commonly practiced in village areas with minimal biosecurity. The poultry products of these chickens are mostly for domestic consumption.

### 2.2. Description of Outbreaks

**Figure 1:**
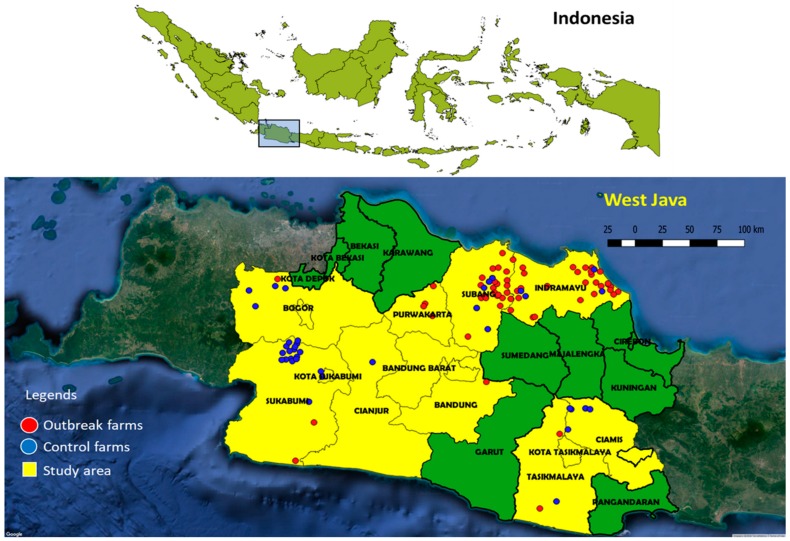
The location of outbreaks and control farms.

From November 2015 to October 2016, outbreak investigations were conducted in seven districts of the West Java province: Bandung, Ciamis, Indramayu, Subang, Sukabumi, Purwakarta and Tasikmalaya (Figure 1). The study was preceded by a pilot study to explore the feasibility of collecting the samples from clinical outbreaks, processing and testing them properly and collecting the information. The selected districts were selected based on HPAI H5N1 history, poultry density, and presence of various types of poultry and poultry production systems. The investigations were conducted in collaboration with the Animal Health Agencies in the districts, who received a financial incentive for confirmed outbreaks.

Poultry farmers reported suspected outbreaks to the district veterinary officers, who subsequently visited the farm. They inspected the poultry for the presence of HPAI associated clinical signs, such as cyanosis and edema of the head, comb, or wattle, respiratory and neurological signs, and lethargy. In addition, the number of sick and dead birds was recorded. In the case that the district veterinary officer considered the farm HPAI suspect, samples were collected. Oropharyngeal samples and cloacal samples of five sick birds were pooled and stored separately in brain heart infusion broth containing antibiotics according to the L237/8 Official Journal of European Union EN 31.8.2006 under chilled conditions. The samples were delivered by the veterinary officers to the local Animal Health Laboratory Cikole of West Java Province, or District Investigation Centre Subang of the Directorate General of Livestock and Animal Health Services, at maximum within 48 hours after they had been collected.

### 2.3. Testing of the Samples

The samples were tested in the Animal Health Laboratory Cikole and District Investigation Center Subang using real-time reverse transcriptase polymerase chain reaction (real-time RT-PCR) targeting the M gene detecting any Influenza A viruses from different hosts including avian, equine and other species. Samples with CT values less than 40 were considered positive for (HP)AIV and used for subtyping by using H5 specific RT-PCRs [24,25,26]. Samples with a CT value < 30 were subjected to Sanger Sequencing at the Eijman Institute for Molecular Biology as described previously [27].

### 2.4. Collection of Epidemiological Data

The location of each poultry farm was recorded at the village level (Figure S2). Poultry type, poultry sector according to Food and Agriculture Organization (FAO) [23,28], open or closed housing, the purpose of raising poultry (commercial/non-commercial) and the number of birds were also recorded during the visit. Visitors to the poultry in the farms were also documented. Furthermore, the date of the first onset of disease according to the farmer, clinical signs presented by the birds and morbidity and mortality were recorded. In addition, the vaccination status was recorded (Figure S2 and Table S1).

Five additional outbreaks detected in the same period in the study region were included; two in sector 4 farms in Sukabumi obtained via the Animal Health Laboratory Cikole and three in farms in Bogor and Sukabumi obtained from ProLab Diagnostic Laboratory, a poultry industry laboratory, to also include sector 1 in the study.

### 2.5. Description of Control Farms

As control farms we selected farms (Table S1) in the study region (to ensure similar exposure to HPAI A(H5N1)) without a history of HPAI from the collected questionnaire (Figure S1). Absence of a poultry farm database comprising farmers from all poultry sectors in West Java made random selection of control farms not possible. To ensure a representative sample of HPAI free farms in the study region local animal health officials with expertise of poultry production in the districts selected farms aiming for a representative sample of the major poultry producing farms in their region. In addition farms were selected from Sierad Produce to also include sector 1 farms from the study region, as they could not be selected by the local animal health officials [29]. From the selected farms we included a total of 60 farms without any history of HPAI A(H5N1) infection.

### 2.6. Data Analysis

Seven categorized factors: District, Poultry Type, Housing System, Sector, Farm Size, Purpose of raising the poultry and number of incoming contacts were included as dependent variables in the data analysis. To prevent imbalance due to small categories districts were grouped in three, namely Indramayu, Subang and Rest. Poultry Type was classified as backyard chickens, ducks and others (broiler breeders and commercial broilers, laying hens, native chickens, quails, and turkeys). Poultry Housing System was grouped as open or closed house; in an open house birds are confined, but the poultry house does not have a closed wall. The purpose of raising was household or commercial and farm size was categorized as less than 1000 birds per farm and 1000 or more birds per farm. The number of visitors were categorized as below or above 10 in the preceding 14 days.

The dataset was compiled in a Microsoft Excel file and transferred to an open-source integrated R environment for statistical analysis and graphics, RStudio (version version 1.1.463, https://www.rstudio.com/).

The association between potential risk factors and HPAI status was determined by logistic regression analysis using outbreak (yes/no) as dependent variable and the potential risk factors mentioned above as independent variables. Data were analysed using a logistic regression model. First, each independent variable was subject to univariate analyses and, subsequently, a full model was made including all independent variables, taking into account collinearity. Next, the variables with the highest p-value were removed from the model stepwise, until the model with the lowest value of Akaike’s information criterion (AIC) was obtained (final model). R package “Stats and MASS, was used for logistic regression and the odd ratio’s (OR’s) were calculated using MASS package and confirmed using mfx package.

## 3. Results

### 3.1. Outbreaks Description

A total of 75 HPAI suspected outbreaks were reported by farmers during the period of this study. In 64 of these suspicions (85%) samples turned out positive in real-time M RT-PCR, indicating a high specificity of the reporting. With the additional five positive farms, as mentioned in the Materials and Methods, a total of 69 outbreak farms are displayed in Figure 1. From 39 of these samples a full genome sequence was obtained as decribed previously [27].

**Figure 2:**
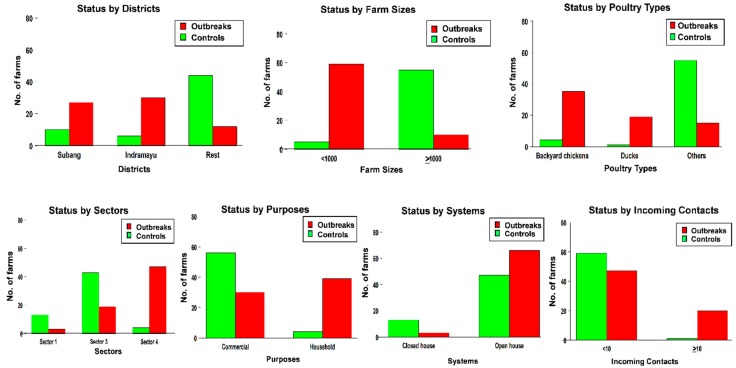
Distribution of outbreak and control farms across districts, farm size, poultry type, sector, purpose, housing systemand incoming contacts.

Most outbreaks were observed in Indramayu (30 farms) and Subang (27 farms). The rest of the outbreaks were located in Purwakarta (four farms), Sukabumi (four farms), Tasikmalaya (two farms), Bandung (one farm), Bogor (one farm). Descriptive results are presented in Figure 2. Backyard chickens (35 farms) and ducks (19 farms) were the dominant poultry types on the outbreak farms. The other affected poultry types were breeders (two farms), commercial broilers (one farm), layers (two farms) and native chickens and quails (10 farms) as displayed in Table S1.

Most HPAI outbreaks were detected on small farms (59 farms housed less than 1000 birds) and most farms had an open poultry house and produced poultry for household purposes. From all total outbreaks, 47 farms were categorized as sector 4, while 19 farms were categorized as sector 3 and three farms belonged to sector 1. Twenty outbreak farms had more than 10 vistors to the farm in the preceding fortnight.

**Figure 3:**
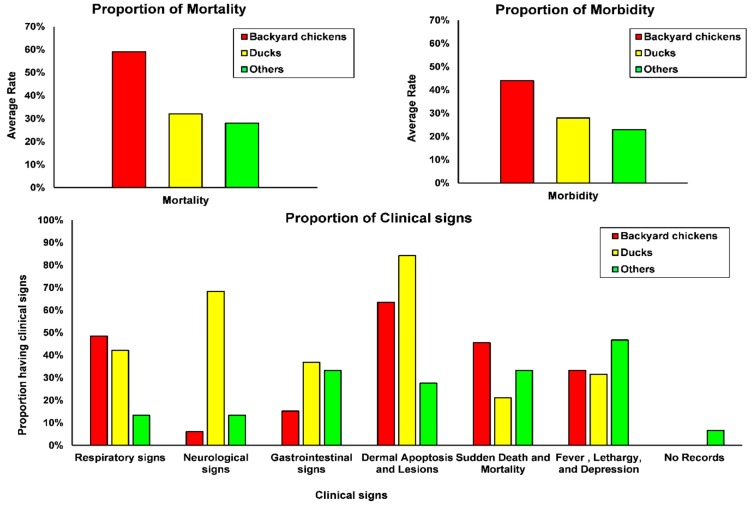
The average mortality and morbidity per farm in the different poultry types and the morbidity associated clinical signs.

The recorded mortality and morbidity are shown in Figure 3. The highest mortality was reported in backyard chickens (average 59%, CI_95%_: 49–69%), compared to ducks (average 32%, CI_95%_: 19–45%) and others (average 28%, CI_95%_: 16–40%). Observed morbidity was also highest in backyard chickens, with average morbidity of 44% (CI_95%_: 32–54%), whereas it was 28% in ducks (CI_95%_: 17–38%) and 23% in others (CI_95%_: 9–37%). Higher mortality than morbidity indicates that a proportion of the birds were found dead before the farmers noticed clinical signs. On four farms poultry had been vaccinated, two farms with broiler breeders, a duck and a quail farm. The observed mortality was 30% on each of the two broiler breeder farms, 50% on the duck and 26% on the quail farm.

The distribution of observed clinical signs over the poultry categories is shown in Figure 3. Dermal apoptosis and lesions (64%, CI_95%_: 52–76%) and respiratory signs (39%, CI_95%_: 27–51%) were overall observed most frequently. Neurological signs were most frequently observed in ducks (68%, CI_95%_: 47–90%). With respect to virus type, H5N1 clade 2.3.2.1c A was associated with more neurological signs than the other viruses, where as H5N1 clade 2.3.2.1c B showed more fever lethargy and depression.

**Figure 4:**
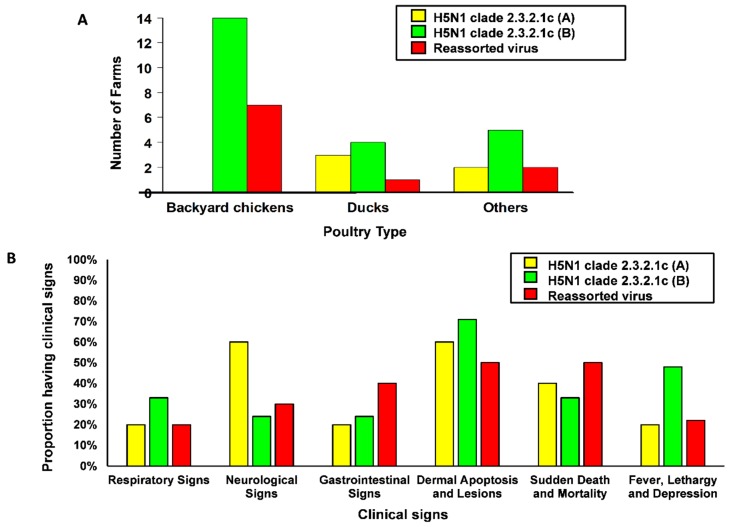
The number of H5N1 clade 2.3.2.1c A and B and reassorted H5N1 viruses in different poultry types (**A**) and the morbidity associated clinical signs in different subgroup H5N1 clade 2.3.2.1c A and B and reassorted H5N1 viruses (**B**) as mentioned in a parallel study [27].

Also H5N1 clade 2.3.2.1c A was predominantly in Duck farms with proportion 60%, (CI_95%_: 14.6–94.7%) while H5N1 clade 2.3.2.1c B (63%, CI_95%_: 40.6–81.2%) and reassorted H5N1 (67%, CI_95%_: 29.9–92.5%) were mostly discovered in backyard chicken farms as displayed in Figure 4. Interestingly the observed clinical signs showed that the neurological signs were also predominantly in Ducks.

### 3.2. Description of Controls

A total of 60 control farms were included in the study (Figure 1) Distribution of potential risk factors across the control farms is shown in Figure 2. The control farms included commercial broilers (36 farms), broiler breeders (seven farms), layers (seven farms), backyard chickens (four farms), ducks (one farm) and native chickens and quails (five farms). Most control farms housed more than 1000 birds (55 farms) and kept poultry for commercial purposes (56 farms) and used an open house system (47 farms) as displayed in Table S1.

### 3.3. Factors Associated with HPAI Outbreaks

In univariate logistic regression all factors were significantly associated with the odds of an HPAI outbreak (Table 1). Multivariate analysis showed, however, that only Poultry Type, Farm Size and incoming contacts were significantly associated with HPAI outbreak. The analysis unveiled that ducks had 15.4 times higher odds of an HPAI outbreak (CI_95%_: 1.12–706) compared to backyard chickens, while other poultry types had a similar odd compared to backyard chickens. Furthermore, larger farms had a much lower odds to get infected than small farms (OR: 0.019, CI_95%_: 0.00095–0.128). Also, farms having at least 10 visitors per fortnight had 13.5 times higher odds (CI_95%_: 1.7–300) than those visited less frequently (Table 1).

## 4. Discussion

The aims of this study were to detect outbreaks of HPAI H5N1 in an endemically infected region, describe their characteristics and identify risk factors associated with these outbreaks. A total of 64 outbreaks were detected, indicating that HPAI A(H5N1) is still endemically circulating in this part of Indonesia, mostly in back-yard chickens and ducks. New findings of the study are the association between incoming contacts and the odds of an HPAI A(H5N1) outbreak and a comprehensive description of the clinical manifestation of outbreaks in various poultry types of the currently circulating virus clade 2.3.2.1c.

In this study, 75 suspected HPAI outbreaks were reported and 64 of these were confirmed (85.3%). This indicates a high specificity of field diagnosis by district animal health officers and community-based reporting. Participatory disease surveillance programs may have increased the ability of poultry farmers to recognize HPAI outbreaks. The higher specificity of clinical diagnosis in this study (85%) than previously observed (29%) [30] also underpins the capability of the district animal health officers to collect samples from clinically suspected animals and it indicates that the samples have been handled and stored correctly before testing. This shows the potential of an HPAI outbreak investigation in Indonesia. In that respect it is notable that the number of detected outbreaks in the study period in the seven districts was much higher than during the years before and after (http://www.oie.int/wahis_2/public/wahid.php/Diseaseinformation/statusdetail). This may have been due to the use of incentives in this study. On the other hand, results of genetic analyses of the samples suggested many undetected outbreaks during the study period [27], indicating that the sensitivity of passive surveillance during the study period was still limited. Vaccination may have contributed to reduced sensitivity of passive surveillance, as it can lead to silent spread of the virus [10]. Nevertheless, the four vaccinated outbreak farms had a clear clinical manifestation of the infection, which could have been due to a mismatch between vaccine and circulating virus or improper vaccination of the flocks.

Overall morbidity and mortality were higher in backyard chickens than in ducks, which may be due to better adaptation of the virus to ducks [31,32,33]. Nevertheless, clinical signs of HPAI observed in the ducks were more prominent than previously reported [31,32]. Neurological signs in particular were observed frequently. Also, dermal apoptosis and lesions were observed frequently in ducks, but also in backyard chicken. The results suggest that clade 2.3.2.1c of HPAI A(H5N1) is more virulent to ducks than the previously circulating clade 2.1.3. Our observations are in line with those observed in ducks after experimental inoculation of this virus clade [34], but the background for the difference in clinical manifestations of the two different subgroup of A(H5N1) clade 2.3.2.1c (A and B) is not clear yet. Previous pathobiological study of A(H5N1) infections showed different clinical manifestation of Indonesian A(H5N1) viruses representing clades 2.1.1 and 2.1.3 in ducks and chickens [35]. The clinical manisfestation upon experimental infection with A(H5N1) viruses representing different groups in clade 2.3.2.1c has not been reported yet.

Ducks have a higher risk of HPAI than other poultry types, which is in agreement with previous studies in Indonesia [36,37,38]. Free ranging ducks in the post-harvest paddy fields can easily encounter AI viruses from wild bird populations or other free ranging ducks. Also, contacts between duck flocks support the transmission of HPAI between duck farms in Indonesia [39].

Farm size was significantly associated with HPAI in this study. The lower risk of HPAI in larger farm size (≥1000 birds per farm) is in contrast with previous studies in Indonesia and a study in Thailand [18,40]. Most likely flock size acts as a confounder in this study for better management practices, better vaccination programs and better biosecurity in large farms than in small ones in West Java [17]. To our knowledge this is the first study showing that an increasing rate of persons visiting the farm is associated with an increased risk of HPAI infection. Consequently, advising farmers to minimize admittance of visitors to their poultry and ensure visitors follow biosecurity protocols are potential measures to reduce HPAI infection. Although Figure 1 shows a clustering of outbreaks across the districts, the factor district was deleted in the stepwise backward selection to get to the final statistical model, because it was not significantly associated with HPAI outbreak. This implies that the significant factors in the final model “poultry type, farms size and incoming contacts” were not evenly distributed across the districts and from that the model concludes that it is actually these factors that explain the difference between outbreak and control farms, and not the districts.

This study has its limitations. Vaccination data of control farms were unavailable so the odds of vaccination as risk factor could not be quantified. The presence of four vaccinated farms among the outbreaks shows, however, that vaccination does not prevent all outbreaks. Participation of farms in the study depended on the willingness of poultry farmers and industry to cooperate, which may have introduced bias. Nevertheless, this study is unique, because it was based on enhanced passive surveillance and structured investigation of suspected outbreaks in a restricted area and time period, which has not been performed in Indonesia before. 

Results of this study indicate that the HPAI A(H5N1) virus is still endemically circulating in West Java. Also vaccinated flocks can be affected by HPAI, indicating poor match between circulating virus and vaccine, or too low vaccination coverage. Further change of the Indonesian poultry production system to bigger farms able to invest in proper biosecurity and cold chain slaughter systems might improve the HPAI situation on the long term [41,42,43,44,45]. Until then, to reduce virus circulation and lower the risk of human exposure increasing vaccination coverage combined with outbreak detection in backyard production systems is required, in addition to reducing the rate of visitors in contact with poultry as demonstrated by this study.

## 5. Conclusions

HPAI A(H5N1) was still endemically circulating in West Java 2015-2016, in particular in ducks and in backyard chickens. Despite the endemic circulation, morbidity and mortality observed in outbreaks are still high, and in ducks neurological signs frequently occur. The rate of persons visiting a farm was associated with the odds of HPAI infection. Results indicate that better external biosecurity might reduce transmission of HPAI A(H5N1) in Indonesia.

## Figures and Tables

**Table 1:** The odds ratio of Outbreak and Control farms with statistical significance univariate and multivariate analysis.

	Factors	Categories	Odds Ratio		
			Estimate	Std.Err	*p*-Value
**Univariate Analysis**	District	Indramayu	Ref [1]		
		Subang	0.54	0.16–1.65	0.28851
		Rest	0.054	0.017–0.15	1.46 × 10^−7^ ***
	Poultry type	Backyard Chickens	Ref [1]		
		Ducks	2.71	0.295–44.103	0.502
		Others	0.031	0.008–0.0923	8.75 × 10^−9^ ***
	Farm size	<1000	Ref [1]		
		≥1000	0.015	0.0044–0.044	5.67 × 10^−13^ ***
	Housing system	Closed house	Ref [1]		
		Open (shed) house	6.085	1.84–27.62	0.00689 **
	Purpose	Household	Ref [1]		
		Commercials	0.054	0.015–0.152	3.86 × 10^−7^ ***
	Sector	Sector 4	Ref [1]		
		Sector 3	0.038	0.0102–0.1082	2.58 × 10^−8^ ***
		Sector 1	0.019	0.003–0.086	1.93 × 10^−6^ ***
	Inbound contacts	<10	Ref [1]		
		≥10	25.106	4.95–458.76	0.002 **
**Multivariate Analysis**	Poultry type	Backyard Chickens	Ref [1]		
		Ducks	15.442	1.12–706	0.082084.
		Others	1.222	0.14–26.58	0.869206
	Farm size	<1000	Ref [1]		
		≥1000	0.0190	0.00095–0.12	0.000503 ***
	Incoming contacts	<10	Ref [1]		
		≥10	13.536	1.65–299.88	0.033523 *

* *p* < 0.1; ** *p* < 0.05; *** *p* < 0.01.

## Supplementary Materials

The following are available online at https://www.mdpi.com/2076-2607/7/9/327/s1.

## References

[1] Sims L.D., Peiris M., Mackenzie J.S., Jeggo M., Daszak P., Richt J.A. (2013). One Health: The Hong Kong Experience with Avian Influenza. One Health: The Human-Animal-Environment Interfaces in Emerging Infectious Diseases: The Concept and Examples of a One Health Approach.

[2] Bridges C.B., Lim W., Hu-Primmer J., Sims L., Fukuda K., Mak K.H., Rowe T., Thompson W.W., Conn L., Lu X. (2002). Risk of Influenza A (H5N1) Infection among Poultry Workers, Hong Kong, 1997-1998. J. Infect. Dis..

[3] WHO Cumulative Number of Confirmed Human Cases for Avian Influenza A(H5N1) Reported to WHO, 2003-2017. https://www.who.int/influenza/human_animal_interface/H5N1_cumulative_table_archives/en/.

[4] Cox N.J., Trock S.C., Uyeki T.M. (2016). Public health implications of animal influenza viruses. Animal Influenza.

[5] Yang L., Zhu W., Li X., Bo H., Zhang Y., Zou S., Gao R., Dong J., Zhao X., Chen W. (2017). Genesis and Dissemination of Highly Pathogenic H5N6 Avian Influenza Viruses. J. Virol..

[6] Chen L.-J., Lin X.-D., Tian J.-H., Liao Y., Ying X.-H., Shao J.-W., Yu B., Guo J.-J., Wang M.-R., Peng Y. (2017). Diversity, evolution and population dynamics of avian influenza viruses circulating in the live poultry markets in China. Virology.

[7] Tosh C., Nagarajan S., Kumar M., Murugkar H.V., Venkatesh G., Shukla S., Mishra A., Mishra P., Agarwal S., Singh B. (2016). Multiple introductions of a reassortant H5N1 avian influenza virus of clade 2.3.2.1c with PB2 gene of H9N2 subtype into Indian poultry. Infect. Genetics Evol..

[8] Wille M., Tolf C., Avril A., Latorre-Margalef N., Wallerstrom S., Olsen B. (2013). Frequency and patterns of reassortment in natural influenza A virus infection in a reservoir host. Virology.

[9] Beerens N., Heutink R., Bergervoet S.A., Harders F., Bossers A., Koch G. (2017). Multiple Reassorted Viruses as Cause of Highly Pathogenic Avian Influenza A(H5N8) Virus Epidemic, the Netherlands, 2016. Emerg. Infect. Dis..

[10] Poetri O.N., Van Boven M., Claassen I., Koch G., Wibawan I.W., Stegeman A., Van den Broek J., Bouma A. (2014). Silent spread of highly pathogenic Avian Influenza H5N1 virus amongst vaccinated commercial layers. Res. Vet. Sci..

[11] Offeddu V., Cowling B.J., Peiris J.S.M. (2016). Interventions in live poultry markets for the control of avian influenza: A systematic review. One Health.

[12] Pfeiffer D.U., Otte M.J., Roland-Holst D., Zilberman D. (2013). A one health perspective on HPAI H5N1 in the Greater Mekong sub-region. Comp. Immunol. Microbiol. Infect. Dis..

[13] Smith G.J., Naipospos T.S., Nguyen T.D., de Jong M.D., Vijaykrishna D., Usman T.B., Hassan S.S., Nguyen T.V., Dao T.V., Bui N.A. (2006). Evolution and adaptation of H5N1 influenza virus in avian and human hosts in Indonesia and Vietnam. Virology.

[14] Kurscheid J., Millar J., Abdurrahman M., Ambarawati I.G.A.A., Suadnya W., Yusuf R.P., Fenwick S., Toribio J.-A.L.M.L. (2015). Knowledge and Perceptions of Highly Pathogenic Avian Influenza (HPAI) among Poultry Traders in Live Bird Markets in Bali and Lombok, Indonesia. PLoS ONE.

[15] Kurscheid J., Stevenson M., Durr P., Toribio J.-A., Kurscheid S., Ambarawati I.G.A.A., Abdurrahman M., Fenwick S. (2017). Social network analysis of the movement of poultry to and from live bird markets in Bali and Lombok, Indonesia. Transbound. Emerg. Dis..

[16] Henning J., Hesterberg U.W., Zenal F., Schoonman L., Brum E., McGrane J. (2019). Risk factors for H5 avian influenza virus prevalence on urban live bird markets in Jakarta, Indonesia-Evaluation of long-term environmental surveillance data. PLoS ONE.

[17] Yupiana Y., de Vlas S.J., Adnan N.M., Richardus J.H. (2010). Risk factors of poultry outbreaks and human cases of H5N1 avian influenza virus infection in West Java Province, Indonesia. Int. J. Infect. Dis..

[18] Leo L., Marius G., Jianmei W., Christina C., Muhammad H., Xiangming X. (2011). Identifying risk factors of highly pathogenic avian influenza (H5N1 subtype) in Indonesia. Prev. Vet. Med..

[19] Pfeiffer D. Assistance in the Geospatial Analysis of HPAI outbreaks in Indonesia. https://www.google.com.hk/url?sa=t&rct=j&q=&esrc=s&source=web&cd=1&ved=2ahUKEwii1JCjpLnkAhXJDaYKHd9tAS0QFjAAegQIABAC&url=http%3A%2F%2Fwww.fao.org%2Fdocs%2Feims%2Fupload%2F199669%2FPfeiffer_Report_Indonesia_2005.pdf&usg=AOvVaw0e6jKK7CO0gsv7W-oHTp2b.

[20] Dharmayanti N.L.P.I., Hartawan R., Pudjiatmoko, Wibawa H., Hardiman, Balish A., Donis R., Davis C.T., Samaan G. (2014). Genetic Characterization of Clade 2.3.2.1 Avian Influenza A(H5N1) Viruses, Indonesia, 2012. Emerg. Infect. Dis..

[21] Smith G.J.D., Donis R.O., World Health Organization/World Organisation for Animal Health/Food and Agriculture Organization (WHO/OIE/FAO) H5 Evolution Working Group (2015). Nomenclature updates resulting from the evolution of avian influenza A(H5) virus clades 2.1.3.2a, 2.2.1, and 2.3.4 during 2013–2014. Influenza Other Respir. Viruses.

[22] Azhar M., Lubis A.S., Siregar E.S., Alders R.G., Brum E., McGrane J., Morgan I., Roeder P. (2010). Participatory Disease Surveillance and Response in Indonesia: Strengthening Veterinary Services and Empowering Communities to Prevent and Control Highly Pathogenic Avian Influenza. Avian Dis..

[23] FAO FAO Recommendations on the Prevention, Control and Eradication of Highly Pathogenic Avian Influenza (HPAI) in Asia. https://www.google.com.hk/url?sa=t&rct=j&q=&esrc=s&source=web&cd=1&cad=rja&uact=8&ved=2ahUKEwi5z4GUqbnkAhXMad4KHY3VATsQFjAAegQIABAB&url=http%3A%2F%2Fwww.fao.org%2Ftempref%2Fdocrep%2Ffao%2F008%2Fae930e%2Fae930e02.pdf&usg=AOvVaw0Ya5LgQdDh_om5LtD43kw1.

[24] Selleck P., Kirkland P. Avian Influenza. http://www.agriculture.gov.au/SiteCollectionDocuments/animal/ahl/ANZSDP-Avian-influenza-AI.pdf.

[25] Heine H., Trinidad L. Rapid Identification and Pathotyping of Virulent IBDV, NDV and AIV Isolates. https://www.google.com.hk/url?sa=t&rct=j&q=&esrc=s&source=web&cd=1&cad=rja&uact=8&ved=2ahUKEwjag9Xwp7nkAhUPFYgKHWDGBXMQFjAAegQIAhAC&url=https%3A%2F%2Fwww.australianeggs.org.au%2Fdmsdocument%2F566-rapid-identification-and-pathotyping-of-virulent-ibdv-ndv-and-ai-isolates&usg=AOvVaw07m4_7rvBBYbq4h9rwr7p0.

[26] Heine H., Foord A., Wang J., Valdeter S., Walker S., Morrissy C., Wong F., Meehan B. (2015). Detection of highly pathogenic zoonotic influenza virus H5N6 by reverse-transcriptase quantitative polymerase chain reaction. Virol. J..

[27] Karo-karo D., Bodewes R., Wibawa H., Artika M., Pribadi E.S., Diyantoro, Pramono W., Sugama A., Hendrayani N., Indasari I. (2019). Reassortments Among Avian Influenza A(H5N1) Viruses Circulating in Indonesia, 2015–2016. Emerg. Infect. Dis..

[28] Rushton J., Viscarra R., Bleich E.G., McLeod A. (2006). Impact of avian influenza outbreaks in the poultry sectors of five South East Asian countries (Cambodia, Indonesia, Lao PDR, Thailand, Viet Nam) outbreak costs, responses and potential long term control. World’s Poult. Sci. J..

[29] Wibawa H., Karo-Karo D., Pribadi E.S., Bouma A., Bodewes R., Vernooij H., Diyantoro, Sugama A., Muljono D.H., Koch G. (2018). Exploring contacts facilitating transmission of influenza A(H5N1) virus between poultry farms in West Java, Indonesia: A major role for backyard farms?. Prev. Vet. Med..

[30] Robyn M., Priyono W.B., Kim L.M., Brum E. (2012). Diagnostic Sensitivity and Specificity of a Participatory Disease Surveillance Method for Highly Pathogenic Avian Influenza in Household Chicken Flocks in Indonesia. Avian Dis..

[31] Henning J., Wibawa H., Morton J., Usman T.B., Junaidi A., Meers J. (2010). Scavenging Ducks and Transmission of Highly Pathogenic Avian Influenza, Java, Indonesia. Emerg. Infect. Dis..

[32] Wibawa H., Bingham J., Nuradji H., Lowther S., Payne J., Harper J., Junaidi A., Middleton D., Meers J. (2014). Experimentally Infected Domestic Ducks Show Efficient Transmission of Indonesian H5N1 Highly Pathogenic Avian Influenza Virus, but Lack Persistent Viral Shedding. PLoS ONE.

[33] Jeong O.-M., Kim M.-C., Kim M.-J., Kang M.-I., Kim H.-R., Kim Y., Joh S.-J., Kwon J.-H., Lee Y.-J. (2009). Experimental infection of chickens, ducks and quails with the highly pathogenic H5N1 avian influenza virus. J. Vet. Sci..

[34] Damayanti R., Wiyono A., Nuradji H., Cahyono M.I. (2017). The pathogenecity of H5N1 highly pathogenic Avian Influenza (HPAI) virus clade 2.3.2. in Indonesian indigenous chicken by contact tranmission with infected duck. J. Indones. Trop. Anim. Agric..

[35] Wibawa H., Bingham J., Nuradji H., Lowther S., Payne J., Harper J., Wong F., Lunt R., Junaidi A., Middleton D. (2013). The pathobiology of two Indonesian H5N1 avian influenza viruses representing different clade 2.1 sublineages in chickens and ducks. Comp. Immunol. Microbiol. Infect. Dis..

[36] Gilbert M., Chaitaweesub P., Parakamawongsa T., Premashthira S., Tiensin T., Kalpravidh W., Wagner H., Slingenbergh J. (2006). Free-grazing ducks and highly pathogenic avian influenza, Thailand. Emerg. Infect. Dis..

[37] Gilbert M., Xiao X., Pfeiffer D.U., Epprecht M., Boles S., Czarnecki C., Chaitaweesub P., Kalpravidh W., Minh P.Q., Otte M.J. (2008). Mapping H5N1 highly pathogenic avian influenza risk in Southeast Asia. Proc. Natl. Acad. Sci. USA.

[38] Loth L., Gilbert M., Osmani M.G., Kalam A.M., Xiao X. (2010). Risk factors and clusters of Highly Pathogenic Avian Influenza H5N1 outbreaks in Bangladesh. Prev. Vet. Med..

[39] Henning J., Pfeiffer D.U., Stevenson M., Yulianto D., Priyono W., Meers J. (2016). Who Is Spreading Avian Influenza in the Moving Duck Flock Farming Network of Indonesia?. PLoS ONE.

[40] Tiensin T., Chaitaweesub P., Songserm T., Chaisingh A., Hoonsuwan W., Buranathai C., Parakamawongsa T., Premashthira S., Amonsin A., Gilbert M. (2005). Highly pathogenic avian influenza H5N1, Thailand, 2004. Emerg. Infect. Dis..

[41] Capua I., Cattoli G. (2013). Prevention and control of highly pathogenic avian influenza with particular reference to H5N1. Virus Res..

[42] FAO-OIE BIOSECURITY FOR HIGHLY PATHOGENIC AVIAN INFLUENZA. https://www.google.com.hk/url?sa=t&rct=j&q=&esrc=s&source=web&cd=1&cad=rja&uact=8&ved=2ahUKEwjQhNifq7nkAhWDMd4KHZAjAI8QFjAAegQIBRAC&url=http%3A%2F%2Fwww.fao.org%2F3%2Fa-i0359e.pdf&usg=AOvVaw0aUv20_f0tyTunoNW5k6_a.

[43] Nastasijevic I., Lakicevic B., Petrović Z. Cold Chain Management in Meat Storage, Distribution and Retail: A Review. https://iopscience.iop.org/article/10.1088/1755-1315/85/1/012022/meta.

[44] World Health Organization, Regional Office for South-East Asia Public Health Interventions for Prevention and Control of Avian Influenza. A Manual for Improving Biosecurity in the Food Supply Chain: Focusing on Live Animal Markets. https://apps.who.int/iris/bitstream/handle/10665/205700/B0237.pdf?sequence=1&isAllowed=y.

[45] FAO LESSONS FROM HPAI. A Technical Stocktaking of Outputs, Outcomes, Best Practices and Lessons Learned from the Fight against Highly Pathogenic Avian Influenza in Asia 2005–2011. https://www.google.com.hk/url?sa=t&rct=j&q=&esrc=s&source=web&cd=1&cad=rja&uact=8&ved=2ahUKEwjt9tqurLnkAhV1IqYKHQRbBtQQFjAAegQIABAC&url=http%3A%2F%2Fwww.fao.org%2F3%2Fa-i3183e.pdf&usg=AOvVaw0xSqXWIbM8bAO3-dyOr4f9.

